# Major challenges to scale up of visual inspection-based cervical cancer prevention programs: the experience of Guatemalan NGOs

**DOI:** 10.9745/GHSP-D-14-00073

**Published:** 2014-07-31

**Authors:** Anita Nandkumar Chary, Peter J Rohloff

**Affiliations:** a Washington University in St. Louis, School of Medicine and Department of Anthropology, St. Louis, MO., USA; b Harvard Medical School, Brigham and Women's Hospital, Boston, MA., USA

## Abstract

**Background:**

Like many other low- and middle-income countries, Guatemala has adopted visual inspection with acetic acid (VIA) as a low-resource alternative to the Pap smear for cervical cancer screening. Nongovernmental organizations (NGOs) introduced VIA to Guatemala in 2004, and a growing number of NGOs, working both independently and in collaboration with the Guatemalan Ministry of Health, employ VIA in cervical cancer prevention programs today. While much research describes VIA efficacy and feasibility in Latin America, little is known about NGO involvement with VIA programming or experiences with VIA outside the context of clinical trials and pilot projects in the region.

**Methods:**

To explore challenges faced by NGOs implementing VIA programs in Guatemala, we conducted semi-structured interviews with 36 NGO staff members involved with 20 VIA programs as direct service providers, program administrators, and training course instructors. Additionally, we collected data through observation at 30 NGO-sponsored cervical cancer screening campaigns, 8 cervical cancer prevention conferences, and 1 week-long NGO-sponsored VIA training course.

**Results:**

Frequently highlighted challenges included staff turnover, concerns over training quality, a need for opportunities for continued supervision, and problems with cryotherapy referrals when immediate treatment for VIA-positive women was unavailable.

**Conclusions:**

Reducing staff turnover, budgeting to train replacement providers, standardizing training curricula, and offering continued supervision are key strategies to improve VIA service quality and program sustainability. Alternative training methods, such as on-the-job mentoring and course prerequisites of online learning, could help increase training time available for clinical supervision. Efforts should be made to ensure that VIA testing is coupled with immediate cryotherapy, that providers trained in VIA are also trained in cryotherapy, and that cryotherapy supplies and equipment are maintained. Where this is not possible and only VIA screening is available, referral systems must be strengthened.

## Abstract (precis)

Scale up of visual inspection with acetic acid (VIA) in Guatemala encountered major challenges, including high attrition of people trained, didactic training without hands-on skills building, lack of continued supervision, and provision of VIA alone without immediate on-site provision of cryotherapy.

## INTRODUCTION

Cytology-based screening can significantly reduce cervical cancer incidence and mortality among previously unscreened populations. In high-income countries, it is very efficacious.^1^ However, in many low- and middle-income countries (LMICs), the availability of cytology-based screening and follow-up is limited. These countries lack infrastructure for transporting and processing cytological samples, performing colposcopy, interpreting biopsies, and providing follow-up care to patients through multiple clinical visits from initial screening to treatment.^2^^,^^3^ As a result, the great majority of the 266,000 deaths worldwide each year due to cervical cancer occur in LMICs.^4^

The great majority of the 266,000 deaths each year due to cervical cancer occur in low- and middle-income countries.

The World Health Organization (WHO) and the Alliance for Cervical Cancer Prevention (ACCP) have promoted visual inspection with acetic acid (VIA) as a low-cost, safe, and effective alternative to cytological screening in resource-poor settings.^5^^,^^6^ When coupled with cryotherapy, VIA allows for a single-visit approach, in which women with precancerous cervical lesions detected during the exam can be immediately treated. Indeed, the “see-and-treat” paradigm has been advanced as the primary advantage of the technique.^7^ VIA and cryotherapy can be performed inexpensively, without electricity, and by specifically trained non-physician providers.^8^ Randomized controlled trials comparing VIA with no screening have demonstrated reductions in cervical cancer incidence and mortality through VIA-based screening.^9^^,^^10^

VIA is becoming a common approach to cervical cancer screening in Guatemala, a small Central American country with a large rural population.^11^ Cervical cancer is the second most common cancer and the second leading cause of cancer deaths among Guatemalan women.^4^ Free health care is a constitutional right for Guatemalan citizens, and the Guatemalan Ministry of Health (MOH) offers women Papanicolaou (Pap) smears in its facilities free of charge.^12^ However, about half of Guatemala's population lives in rural areas^11^ with limited access to health care, including cytological screening.^12^ Annual screening coverage by the MOH is only 12%–18%.^13^ Total screening coverage of women ages 25–64 in Guatemala is estimated at 40%,^14^ in contrast to WHO recommendations of 80% coverage for successful national screening programs.^6^ Furthermore, cytology services are centralized in urban areas, quality control is poor, and delayed reporting of results and loss of patients to follow-up is common.^15^^–^^17^ Vaccination against human papillomavirus (HPV), which causes most cervical cancer, and HPV DNA testing are not yet available through public-sector care.^13^

In this context, nongovernmental organizations (NGOs) have played an important role in increasing cervical cancer screening coverage. As a result of health care reform in the 1990s, financed by the Inter-American Development Bank and the World Bank, many basic health services in Guatemala are now delivered through government-contracted NGOs.^18^^,^^19^ Many NGOs also work independently of the MOH to deliver health care services to impoverished populations. There are an estimated 10,000–15,000 NGOs currently operating in Guatemala,^20^ with an estimated 45%–60% offering some level of health care programming.^21^^–^^23^

Cervical cancer screening and treatment became priorities for many NGOs in the last decade. NGOs provide at least 15% of all cervical cancer screening nationally and a much larger percentage in rural areas.^13^ One of the NGO sector's most significant contributions to cervical cancer prevention in Guatemala has been pioneering the use of VIA–cryotherapy. In 2004, in an effort to overcome problems with cytology-based screening, a small group of NGOs began pilot-testing VIA–cryotherapy in their own clinics. These NGOs also approached local MOH administrators in 3 of the 24 MOH-designated national health districts, who agreed to have staff at selected clinics participate in NGO-sponsored VIA–cryotherapy training and screening campaigns monitored and evaluated by the NGOs.^24^ Success with initial demonstration projects encouraged the development of several formal NGO-sponsored VIA–cryotherapy programs in various regions throughout the country.

One of the NGO sector's most significant contributions to cervical cancer prevention in Guatemala has been pioneering the use of VIA–cryotherapy.

In part due to NGO leaders' advocacy and NGO-sponsored VIA trainings for MOH staff, MOH officials incorporated VIA into national health care services in 2008. The MOH now has its own national VIA program: Officials report training thousands of providers in VIA or VIA–cryotherapy since 2008 and estimate that approximately two-thirds of all MOH-conducted cervical cancer screening is VIA-based.^13^ For several years, both before and during MOH integration of VIA into the national reproductive care program, NGOs constituted the primary trainers of VIA providers in Guatemala. Leaders of the most prominent NGOs that offer training courses have certified at least 1,000 NGO and MOH providers in VIA or VIA–cryotherapy (personal communication with Patty Baiza, Clinical Coordinator, Faith in Practice, May 2013; Ana Garcés, Director, Una Voz Contra El Cáncer, July 2012).

Today, many NGOs continue to offer training courses for their own staff, foreign volunteers, lay health promoters, lay midwives, and MOH physicians and nurses. As well, many NGOs continue to operate VIA-based cervical cancer prevention programs. Some organizations work independently of the MOH, holding VIA screening campaigns or including VIA in broader reproductive or primary health care services. Other NGOs offer a variety of VIA support services such as donating materials for VIA exams and cryotherapy equipment to MOH facilities and other NGOs; providing training courses to MOH personnel and staff of other NGOs; continually supervising MOH or NGO trainees; and offering cryotherapy treatments for women referred from MOH facilities or NGOs lacking cryotherapy equipment.

While much research has been conducted about the efficacy of VIA and VIA training courses in Latin America,^25^^–^^29^ little is known about VIA implementation outside of clinical trials or about the attitudes of providers in the region toward the technology.^30^ In Guatemala, NGOs have been at the center of VIA service implementation and training of providers for over a decade. As VIA is increasingly incorporated into national health policies throughout Latin America, it is important to understand the experiences that these organizations have had with VIA-based cervical cancer screening and with providing training and capacity building to MOH facilities.

## METHODOLOGY

### Study Design

We identified 20 NGOs operating VIA programs in Guatemala through online NGO directories, investigator participation at cervical cancer prevention conferences, and local recommendations by MOH and NGO staff. As there is no comprehensive NGO registry in Guatemala, it is not possible to determine the proportion of NGOs with VIA programming reflected in the sample. However, the NGOs identified do represent the full spectrum of program offerings in terms of: (a) types of VIA programming (direct service provision and/or training course); (b) models of service provision (one-time community campaigns, established VIA clinics, or screening as part of a comprehensive health care program); (c) geographical scope of programming (working in one department, multiple departments, or countrywide); and (d) collaboration with the MOH (working independently from or in collaboration with the MOH). Table 1 describes NGO characteristics. Table 2 describes models of NGO collaboration with the MOH.

**Table 1.** Characteristics of NGO Sample

**Characteristic**	**Number**
**Organizational Focus** (n = 20)	
Cervical cancer screening	3
Maternal–child health	1
Medical clinic	3
Medical/surgery mission trips	4
Primary health care	2
Reproductive health	7
**Type of Program** (n = 20)	
Service provision	13
Service provision and training course	4
Training course	3
**Service Provision Model** (n = 17)	
Community campaigns	6
Comprehensive care	7
Comprehensive care and community campaigns	1
VIA clinic	3
**Collaboration With MOH** (n = 20)	
Yes	11
No	9
**Region** (n = 20)	
Central Highlands	6
Central Highlands and Pacific Coast	1
Countrywide	6
Pacific Coast	1
Peten	1
Peten and Verapaces	1
Western Highlands	3
Western Highlands and Verapaces	1

Abbreviations: MOH, Ministry of Health; NGO, nongovernmental organization; VIA, visual inspection with acetic acid.

**Table 2.** Types of NGO Collaboration With MOH^a^

**Collaboration**	**Number**
Accept cryotherapy referrals from MOH	8
Donate material/equipment to MOH facility	5
Organize campaigns with or in MOH facility	8
Supervise MOH personnel after training course	2
Train MOH personnel	6

Abbreviations: MOH, Ministry of Health; NGO, nongovernmental organization.

a Some NGOs collaborate with the MOH in multiple forms; N = 11 NGOs.

### Data Collection

From May 2012 through August 2013, we conduced semi-structured interviews with 36 staff members of 20 NGO-based VIA programs. We interviewed direct service providers, VIA program administrators, NGO directors, and VIA training course instructors (Table 3). Interviews lasted from 40 minutes to 2 hours and were conducted at the interviewee's workplace in Spanish or English according to the interviewee's preference. We recorded all interviews, with permission, and transcribed them verbatim. In addition, we observed 30 days of screening campaigns and attended 8 cervical cancer prevention conferences and a 1-week VIA training course.

**Table 3.** Characteristics of Interviewees (N = 36)

**Characteristic**	**Number**
**Professional Role in NGO**^a^	
NGO administrator or director	13
Service provider	19
Training course instructor	10
VIA program director	3
Women's health program director	3
**Nationality**	
European	2
Guatemalan	17
Other Latin American	1
U.S.	16
**Professional Training** (place of training)	
Auxiliary nurse (Guatemala)	4
Professional nurse (Guatemala)	5
Nurse practitioner (United States)	3
Physician (n = 8, Guatemala; n = 4, United States)	12
Bachelor's degree	6
Master's degree	6

Abbreviations: NGO, nongovernmental organization; VIA, visual inspection with acetic acid.

a 12 of 36 interviewees held multiple professional roles.

### Data Analysis

We applied an inductive approach to analyze the data.^31^ As common themes emerged through preliminary review of verbatim transcripts and notes from observations, we developed a codebook. Data were coded for dominant themes using the qualitative data analysis software Saturate App. Subsequently, we compared dominant themes by NGO program type (service delivery or training course), engagement with the MOH (collaboration or independent work), and professional identification of the interviewee (service provider, administrator, or training course instructor), as we hypothesized that experiences might differ among these categories. However, in the end, common themes arose with interviewees across all professional roles, program types, and levels of collaboration with the MOH, and so results are not organized by these categories.

### Ethics

The Institutional Review Board of Washington University in St. Louis, MO, approved this research. Locally, NGO administrators granted permission for staff to participate in the study. Interviewees received explanations about the study objectives and assurance of confidentiality. Interviewees then provided verbal consent for participation in the research.

## RESULTS

Interviewees described 4 major challenges in VIA implementation:

- Staff turnover
- Training quality
- Continued supervision
- Cryotherapy referrals

### Staff Turnover

Twenty-four interviewees working with 13 of 20 NGOs reported turnover of staff trained in VIA was a problem—either among former trainees or their own programs' practitioners. Specific concerns with staff turnover differed based on interviewee involvement with training versus service provision.

**Turnover of trainees in the MOH.** NGOs offering training courses encountered difficulties with turnover of trainees, and especially MOH trainees. Central American Ministries of Health rotate personnel between services every few years, particularly with electoral changes in political leadership. NGO program directors reported that MOH nurses they had trained in VIA offered the exam in their posts in reproductive health for 1–2 years but then were rotated into a new service with no VIA component (for example, child nutrition, labor and delivery). Staff turnover within the MOH also results from changes of administration, which can lead to supervisors' decisions to lay off personnel performing VIA and reduce or eliminate VIA program budgets. As one VIA training course instructor reported:

[The MOH] would send us women, nurses, doctors [they] wanted us to train. We trained them. We even gave some of them [cryotherapy] guns. But inevitably, more than half of them, the doctor left to go somewhere else, the nurse got fired, or whoever was in charge of the department, their interest disappeared.

This instructor estimated that only about 50% of the nurses and physicians that his NGO trained in VIA ended up using the skill after the course. Program administrators of another NGO, which had performed a study with former trainees, noted that fewer than one-third of the MOH nurses they had trained over 4 years (2008–2011) were still practicing VIA in 2012. Indeed, the MOH reported that, in 2008, only 50–85 of 1,211 personnel previously trained in VIA (about 4%–7%) still actively provided the service.^32^

One Guatemalan training course instructor feared that lower-level providers would lose their clinical observation skills if not in constant practice:

During the course, many people are trained, and afterwards, they rotate through other services and forget it. If you don't use the skill and just do Pap smears, you will not be able to do [VIA] because it's hard to do. It's often hard to see.

Staff turnover results in temporary or permanent discontinuation of VIA programs when posts remain unfilled. As a result, training course instructors felt that monetary and human resource investments were being used sub-optimally.

Staff turnover results in temporary or permanent discontinuation of VIA programs when posts remain unfilled.

Staff turnover among MOH administrators also disrupted long-term VIA collaborations with NGOs. A Guatemalan program administrator stated:

We made contacts with government officials to train people from the MOH. We began very well and all, but the lines of communication break pretty easily with them. They change personnel every 4 years, every time there is another election, and then whatever you've been doing with them goes downhill.

Some interviewees expressed frustration over expending resources on travel and meetings with MOH representatives when VIA collaborations fell through due to MOH leadership changes. Other interviewees, however, reported building relationships successfully in 2 departments, whose health directors expressed great enthusiasm for VIA and popularized the method among other staff and their successors.

**Turnover in NGOs.** Changes in leadership and staff turnover frequently affected not only NGO–MOH training collaborations but also internal NGO VIA programs. NGO programs were particularly vulnerable to unexpected shifts in funding streams and strategic mission. One European VIA provider voiced frustration that the NGO she worked for tended to “take on a lot of projects” but not follow through with them. VIA became one such project, and shortly after sending her to training, administrators had a “change of heart”: “They decided not to do VIA-cryo for no apparent reason.”

Job shopping and leveraging salary differentials were also major sources of VIA staff turnover in NGOs. In Guatemala, NGOs and the private sector offer health practitioners higher salaries than the public sector,^33^ and salaries vary widely between NGOs of differing sizes. As a result, “internal brain drain” is a common problem. One NGO administrator observed that MOH nurses trained in VIA market this skill and leave their government posts to earn higher wages elsewhere. Similar job switching from less to more lucrative NGO posts was also common. As one NGO administrator from the United States related:

Right now, we are not doing VIA. This was not a strategic decision, but rather bad happenstance. There used to be a community health worker who did community screenings. She got trained in [VIA], and between her and another doctor who got trained in the method, they were able to bring people in. Unfortunately, we lost both staff members [to other NGOs], and so we lost capacity to offer the programming.

Several interviewees remarked that funding constraints prevented them from responding to this “brain drain,” since they had not budgeted to send new staff to VIA training courses. In contrast, one NGO director reported accounting for staff turnover in yearly programming budgets: “That's not a problem for us. We pay to train new providers.”

### Training Quality

Twenty-two interviewees working for 11 of 20 NGOs described ensuring VIA training quality as a serious challenge. Interviewees endorsed the quality of VIA courses delivered by 2 large and well-known NGOs, as well as those offered in some health districts of Guatemala with strong leadership among local MOH administrators. These courses rigorously adhered to VIA educational materials developed by Jhpiego, the organization that designed and pioneered the global gold standard of VIA training courses.^8^

Commonly, however, interviewees described visiting or participating in shorter courses that offered only didactic training with no clinical supervision. For example, while MOH officials at the national level report offering week-long courses,^14^ an interviewee with a part-time MOH affiliation alleged:

[The MOH] was trying to form courses, but with many deficiencies…. [They] offer a 2- or 3-day course that is purely theoretical, with no practicum.

Shorter courses that offer only didactic training, with no clinical supervision, are common.

Similarly, an NGO physician and former MOH employee noted that the MOH scheduled its courses with so little advance notice that participants did not receive or review materials prior to training. Furthermore, she reported, the MOH did not recruit enough women to serve as screening subjects for the training courses, with the result that trainees lacked opportunities for clinical training.

Also, some NGOs and short-term medical missions use non-standard curricula. A Guatemalan nurse related that she had participated in 2 VIA training courses sponsored by U.S. physicians. The first was with an NGO that offered a one-time 3-day VIA course and another with a well-established NGO that had been offering 5-day courses following Jhpiego standards for several years. She contrasted her experiences in the 2 courses:

In the first one, they just told us how to do it, and then we went off on our own and attended the women, but [the instructors] were not there with us. The second time, they were there with us, and it was harder because there were exams.

Because of variations in the length and perceived quality of VIA training courses, several interviewees feared that deficient training might result in low-quality services and negative publicity surrounding VIA. “The problem is that even those who do not pass the course can go on to practice VIA, because there is no regulation to stop them,” a Guatemalan training course instructor lamented. An NGO director from the United States described receiving reports from a relatively small and recently formed North American NGO in eastern Guatemala that had experienced both high false positive and false negative rates in a VIA pilot program. This group, she explained, had offered its practitioners minimal and inadequate training before beginning to offer services:

I'm afraid that some of these smaller groups are out there doing VIA, but there's not any quality control, and they aren't doing it well. And that worries me, because that can give a bad name to the procedure … Pretty soon people are going to say, “Well, that procedure doesn't work.”

### Continued Supervision

Twenty-two interviewees working for 11 of 20 NGOs reported concerns about a lack of continued on-site supervision and long-term support for health care providers after VIA training. Most NGOs and MOH trainers lack resources to offer continued mentoring to former trainees. One training course instructor, a Guatemalan physician, expressed concerns about this as she described visiting a group of Guatemalan nurses and health promoters who had been certified in VIA by a one-time U.S.-sponsored medical mission. These VIA providers had difficulty distinguishing between positive and negative exams, but they did not have the means to communicate with their international trainers for ongoing guidance.

Interviewees described a lack of continued mentoring for MOH-trained providers, as well. A Guatemalan nurse and course instructor reported witnessing problems at government-run VIA clinics, such as examiners using inadequate light sources or no light source at all during exams, sniffing acetic acid to determine its concentration, manipulating the cervix with tongue depressors that obstructed their vision, and performing cryotherapy incorrectly.

Pointing out the implications of such lack of oversight, several interviewees drew attention to an incident in which a group of women were severely burned by undiluted acetic acid during VIA exams gone awry in Guatemala City.^34^ As a Guatemalan VIA training course instructor remarked:

Now [the MOH is] having to remove staff that they trained in VIA from their posts, because this year complications arose. In some public clinics in Guatemala City, they burned patients because they used pure acetic acid without diluting it … So people lost faith; they don't go to that health center anymore.

A U.S. physician working for another NGO reported that, because of this incident, some of her patients resisted VIA exams, which they referred to as “acid Paps,” and demanded “real Paps.”

Program directors often asserted that their own staff could benefit from continuing supervision and refresher courses, as over time some employees had strayed from protocol. For example, some administrators said that their staff performed cryotherapy on women under the age of 25, even though it is recommended that the test be performed only on women ages 25–50.^35^^,^^36^ Additionally, one U.S. program director found that trained local staff treated women with “a little touch” (*toquecito*) of the cryotherapy probe rather than following guidelines to freeze, thaw, and refreeze lesions for 3, 5, and 3 minutes, respectively^35^:

Sometimes I wonder what we're doing. It's like, are you kidding me? Someone gave a 22-year-old a “little touch” of cryo? What does that mean? What does that do? What are we doing? Okay, so now we're doing “little touches” of cryo to all these 20-something-year-old Guatemalan women?

This program director subsequently sent local staff to a refresher course.

### Cryotherapy Referrals

Seventeen interviewees working for 12 of the 20 NGOs mentioned concerns about contact with MOH facilities or other NGO programs in which VIA and cryotherapy are not performed together, within a single visit. As one program director stated:

VIA is not being done here in the way it is proposed in international medical journals. VIA is supposed to be see-and-treat.

In part, this is because some MOH facilities and NGOs in Guatemala have materials to perform VIA exams, but they lack equipment or run out of supplies to perform cryotherapy. While the MOH does not report problems with gas supply at the national level,^32^ interviewees explained that local-level MOH facilities in which providers had been trained in VIA-cryotherapy often lack funds for replenishing gas supplies. Furthermore, there is no national system for maintenance of cryotherapy equipment.^32^

For various reasons, many women who are found to be positive on VIA do not receive cryotherapy immediately, as the see-and-treat paradigm calls for.

**Training in VIA but not cryotherapy.** Another reason that VIA and cryotherapy are not coupled is that some providers are trained in VIA but not cryotherapy. For instance, in 2008, the MOH reported that only 252 of 1,211 VIA-trained providers (about 21%) were also trained in cryotherapy.^32^ Similarly, some NGOs reported certifying only a subset of VIA trainees in cryotherapy. Training course instructors aim to supervise trainees during 5–10 cryotherapy procedures, in line with Jhpiego recommendations.^35^ This generally limits the number of cryotherapy trainees to 1 to 2 practitioners per 100 women screened. However, NGO administrators sometimes enroll more students (for example, 10–20) in their courses than can be trained in cryotherapy, feeling that they can benefit from learning VIA alone until they are eventually trained in cryotherapy in a subsequent course. As a result, about 20%–25% of trainees participating in these courses received training in both VIA and cryotherapy. Not all interviewees agreed with this strategy. One U.S. NGO director familiar with large training courses stated, “I'm scratching my head wondering why they would train you in one and not the other.” This NGO had limited the size of its course to 5 trainees so that all would have the opportunity to learn cryotherapy.

Because of shortages of cryotherapy equipment and trained personnel, NGO and MOH providers may refer women who test positive to other NGOs or larger MOH facilities for treatment. For example, one NGO director reported forming a relationship with the MOH:

The MOH, they're doing VIA, but they don't have the capability to do cryo, so they're referring cryotherapy patients to us.

NGO staff also made contact with other NGOs to overcome inability to offer cryotherapy. As one NGO administrator described it:

We did the screening, but what are we going to do to help them [screening-positive women]? We made an alliance with [another NGO]. They give us treatment when we refer patients directly to them … And that's how we're working, because we haven't been trained to do the VIA and the cryotherapy.

**Loss to follow-up.** Nine interviewees working in 7 of 10 NGOs participating in cryotherapy referral networks reported challenges with ensuring follow-up care for VIA-positive women. While interviewees regarded the emergence of NGO–MOH referral networks as a practical solution to resource scarcity, they highlighted 2 major problems with these systems. First, VIA-positive women often did not complete cryotherapy referrals because of economic, transportation, and childcare barriers. One Guatemalan NGO auxiliary nurse, trained in VIA but not cryotherapy, described the case of a woman she had referred for a later cryotherapy appointment at the same facility:

We gave her an appointment so that she would come back to the health center to get her cryotherapy. But she didn't come. So what I was doing was calling and calling … I told her that when she comes, they're going to give her the treatment free, because she'd have spent money on getting there. And she says, “Yes, I'm going to go.” But she doesn't come.

Interviewees who received cryotherapy referrals reported the same problems. At one VIA–cryotherapy campaign we attended, only 2 of 16 women who had been referred for cryotherapy after prior VIA testing arrived for their appointments. The nurse charged with their treatment remarked, “This makes us feel as if we are not really doing anything [with VIA].” A program administrator, exasperated with the situation, stated: “If you can't follow-up on the whole package, I'm not sure how much value there is [in doing VIA].” Some NGOs overcame referral problems by directly organizing and financing transportation for women to complete referrals or working with local MOH facilities and municipal offices to do so.

**Coordination problems.** A second concern with cryotherapy referrals related to the difficulties of inter-institutional coordination. One NGO director reported that her organization had the resources to offer free treatments to women in need of follow-up but had troubles establishing reliable contacts with MOH personnel and staff of another NGO with “broken” cryotherapy equipment willing to coordinate referrals. Rather, many VIA-positive women learned of her clinic on their own, through word-of-mouth. This problem was not as salient in other health districts, where NGOs had established strong relationships with active MOH leaders.

## DISCUSSION

This research identified 4 major challenges faced in NGO-sponsored VIA programs in Guatemala (staff turnover, non-standard training courses, lack of on-site supervision, and disruption of the VIA–cryotherapy “see-and-treat” paradigm), which points to strategies for improving service delivery, sustainability, and resource allocation.

Interviewees most frequently reported **staff turnover** as an important challenge. Because of MOH service rotation systems and competitive NGO hiring practices, VIA-trained providers may not continue to practice the skill. On the positive side, providers who move to other positions take with them increased understanding of VIA–cryotherapy and cervical cancer. On the negative side, however, staff turnover raises concerns about service quality, gaps in service delivery, and suboptimal use of limited training resources, as documented in other countries such as Mozambique, Peru, and Uganda.^37^^,^^38^ Additionally, VIA training may become a marketable skill for MOH providers who leave their posts for higher-paying civil-sector jobs. Such drains of public-sector health care providers by the NGO sector are common in LMICs and by no means limited to VIA training.^39^ This study also indicates that turnover among MOH and NGO administrators can detrimentally affect VIA program continuity. This phenomenon has also been documented in Bolivia, where frequent leadership changes among MOH staff decrease possibilities for VIA-related capacity building.^40^

**Reducing turnover.** While reconsideration of MOH staff rotation systems is advisable in Guatemala, staff turnover is unlikely to change because obligatory staff rotations are a long-standing human resources policy within many Central American Ministries of Health. NGOs operating their own VIA-based screening programs, however, may be able to partially mitigate the effects of staff turnover within the NGO sector and ensure continuity of services by budgeting to train new providers to replace those who leave. Also, NGOs should avoid using salary differentials to hire VIA-trained staff away from either the public sector or other NGOs.^39^ In 2013, the MOH, several major NGOs, and local universities formed a Cervical Cancer Consortium to develop national cervical cancer prevention policy. The Consortium could provide a valuable forum for addressing staff turnover and “internal brain drain” by establishing an “NGO code of conduct,” as has been proposed for the African region,^39^ and setting agreed salary levels for VIA providers.

The Cervical Cancer Consortium could address staff turnover and “internal brain drain” by establishing an “NGO code of conduct” and setting agreed salary levels.

Two additional and interrelated concerns raised by interviewees were **non-standard training courses**, which lacked practice under clinical supervision, and **lack of continued on-site supervision** for former trainees. Interviewees felt that these problems lead to considerable heterogeneity of practice among VIA providers, especially regarding the age range of patients screened and standardized application of cryotherapy. These problems compromised the technique's reputation among local service users and providers, a finding also reported in India.^41^ Program administrators and providers in Guatemala described a need for continued supervision and refresher courses, a perception widely reported in evaluations of VIA programs worldwide.^8^^,^^32^^,^^38^^,^^40^^,^^42^

**Regulating training.** Greater regulation of training programs is necessary. One of the future tasks of the Cervical Cancer Consortium in Guatemala will be standardizing a VIA training curriculum based on Jhpiego materials. A certification system should be developed that allows only those who have passed approved training courses to conduct VIA–cryotherapy. Additionally, both the MOH and NGOs should budget for trained supervisors who can rotate among former trainees to provide guided supervision, identify problems, and assess needs for refresher training.^8^ The Consortium could play a valuable role in setting standards of quality care, ratios of field supervisors to providers, and frequency of on-site mentoring and evaluative visits.

A certification system should be developed that allows only those who have passed approved training courses to conduct VIA–cryotherapy.

**Making time for clinical supervision in training.** Additionally, other training options should be explored. Rather than relying on concentrated training with a minimal period of clinical supervision, VIA training could take place through continuous on-the-job mentoring. Online training modules could serve as pre-requisites for training courses, which could help increase time available for clinical supervision. Similarly, refresher courses could include online components with image and protocol reviews. The Consortium is ideally positioned to develop standardized criteria for such training experiences.

Another major challenge, and the most significant finding of this study, is the endemic **disruption of the VIA–cryotherapy “see-and-treat” paradigm**. Interviews suggested that often only VIA is performed and VIA-positive women are lost to follow-up care. Often, access to functioning cryotherapy equipment is limited, especially in MOH facilities, and many providers are trained only in VIA, but not in cryotherapy. Resource shortages leading to the breakdown of the VIA–cryotherapy link also have been described in other LMICs.^32^^,^^43^

The most significant finding of this study is the endemic disruption of the VIA–cryotherapy “see-and-treat” paradigm.

This situation reflects problems common to all screening and referral models. It is a particularly important problem in this instance because one of the most compelling justifications for VIA–cryotherapy is that providing immediate treatment for VIA-positive patients eliminates financial, administrative, and logistical barriers that lead to loss to follow-up in resource-poor settings. These strengths of the “see-and-treat” paradigm are thought to overcome the shortcomings of cytology-based screening, which has failed to produce significant gains against cervical cancer in LMICs.^7^^,^^15^^,^^17^^,^^44^ Indeed, large trials of VIA-only programs, where VIA is not directly coupled with cryotherapy, have shown problems with follow-up similar to those encountered in cytology-based programs.^2^^,^^45^ Therefore, the VIA-only model is particularly concerning in countries with inadequate and underdeveloped referral systems such as Guatemala.

**Re-coupling VIA and cryotherapy.** The VIA–cryotherapy linkage can be improved through long-range plans for servicing and distributing cryotherapy equipment and comprehensive training programs that certify all providers in both techniques. For the time being, however, it is likely that screening-only VIA programs will proliferate in Guatemala and elsewhere, given the issues of turnover, equipment maintenance, and training capacity documented here. Therefore, VIA funders and implementers working in this context should address the empirical barriers to effective patient referral.

Improving referral systems in LMICs is a complex and ambitious task, but initial steps can be taken to decrease loss to follow-up of VIA-positive women in Guatemala. Mapping of clinical centers offering VIA-only versus VIA–cryotherapy in each health district could facilitate inter-institutional coordination of VIA referrals. Additional actions must be taken to address patients' economic and logistical barriers to obtaining treatment. This study indicates that NGO and MOH clinics may be able to help overcome referral problems by organizing and subsidizing transportation for VIA-positive women to cryotherapy appointments. Additionally, by analogy with successful care coordination systems developed in other LMICs for other conditions, Guatemala's robust national cell phone infrastructure could be harnessed to create shared mHealth platforms for tracking patients and coordinating referrals for VIA-positive women.^46^^,^^47^

Clinics may be able to help overcome referral problems by organizing and subsidizing women's transportation to cryotherapy appointments.

### Limitations

This study has 3 primary limitations. First, as we relied on a convenience sample of NGOs, results may not be generalizable to other NGOs operating VIA programs in Guatemala or in Latin America. However, because the NGOs involved varied widely in terms of size, program models, and extent of collaboration with the MOH, the study likely describes the full range of challenges in NGO-sponsored VIA programming in Guatemala. Second, since the study focused exclusively on NGOs' roles in VIA programs, MOH personnel were not interviewed. Undoubtedly, MOH personnel would describe additional challenges not discussed here. This is an area for future research. Third, given the qualitative nature of the study, we cannot specify the absolute frequency of reported phenomena such as the breakdown of the “see-and-treat” paradigm. Further quantitative research is needed to assess this issue.

## Notes

**Peer Reviewed**

**First published online:** 2014 Jul 31
